# *impresso* Text Reuse at Scale. An interface for the exploration of text reuse data in semantically enriched historical newspapers

**DOI:** 10.3389/fdata.2023.1249469

**Published:** 2023-11-03

**Authors:** Marten Düring, Matteo Romanello, Maud Ehrmann, Kaspar Beelen, Daniele Guido, Brecht Deseure, Estelle Bunout, Jana Keck, Petros Apostolopoulos

**Affiliations:** ^1^Digital History & Historiography, Luxembourg Centre for Contemporary and Digital History, Esch-sur-Alzette, Luxembourg; ^2^Institute of Archeology and Classical Studies (ASA), University of Lausanne, Lausanne, Switzerland; ^3^DHLAB, École Polytechnique Fédérale de Lausanne (EPFL), Lausanne, Switzerland; ^4^Digital Humanities Research Hub, School of Advanced Study, University of London, London, United Kingdom; ^5^Digital Research Infrastructure, Luxembourg Centre for Contemporary and Digital History, Esch-sur-Alzette, Luxembourg; ^6^Royal Library of Belgium, Brussels, Belgium; ^7^Contemporary History of Luxembourg, Luxembourg Centre for Contemporary and Digital History, Esch-sur-Alzette, Luxembourg; ^8^German Historical Institute Washington, Washington, DC, United States

**Keywords:** text reuse, historical newspapers, user tasks, scalable reading, data visualization, comparison, semantic enrichment, *impresso*

## Abstract

Text Reuse reveals meaningful reiterations of text in large corpora. Humanities researchers use text reuse to study, e.g., the posterior reception of influential texts or to reveal evolving publication practices of historical media. This research is often supported by interactive visualizations which highlight relations and differences between text segments. In this paper, we build on earlier work in this domain. We present *impresso* Text Reuse at Scale, the to our knowledge first interface which integrates text reuse data with other forms of semantic enrichment to enable a versatile and scalable exploration of intertextual relations in historical newspaper corpora. The Text Reuse at Scale interface was developed as part of the *impresso* project and combines powerful search and filter operations with close and distant reading perspectives. We integrate text reuse data with enrichments derived from topic modeling, named entity recognition and classification, language and document type detection as well as a rich set of newspaper metadata. We report on historical research objectives and common user tasks for the analysis of historical text reuse data and present the prototype interface together with the results of a user evaluation.

## 1. Introduction

Text reuse detection (TRD) is a powerful technique to identify “meaningful reiteration[s] of text, usually beyond the simple repetition of common language” (Romanello et al., 2014). TRD identifies repeated text segments (or *passages*) and groups them automatically into text reuse *clusters*. In the domain of Digital Humanities research, TRD is often used to trace quotations, allusions, and paraphrases. Not only the presence but also the frequency of reuse can be meaningful: the frequency with which a text is quoted by later authors, for example, serves as a proxy for literary or scholarly reception. A popular example of research enabled by TRD is on historical newspaper collections, as demonstrated by projects such as Oceanic Exchanges (Oiva et al., 2020; Keck et al., 2022) and Viral Texts (Cordell, 2015). In both cases, TRD helped to capture prevalent journalistic practices, such as the repurposing and editing of content, or news phenomena, such as the viral circulation of content.

The project “*impresso*–Media Monitoring of the Past” (2017–2020)^1^ detected text reuse within a corpus of Swiss and Luxembourgish newspapers alongside other forms of semantic enrichment (e.g., topic modeling, named entity recognition, image similarity detection, the detection of content type and language).^2^ Its corpus consists of 76 newspapers published in French, German, and Luxembourgish between 1738 and 2018 and contains ca. 50 million content items^3^ detected in 5.5 million pages. The *impresso* application^4^ supports historians and other humanities researchers with powerful search, filter, and discovery functionalities for the exploration of the enriched data. It is generic in the sense that it supports a wide variety of different use cases. This includes, for example, advanced search, the visualization-aided comparison of large user-generated article collections, and the creation of research datasets for further processing outside the application. In addition, *impresso* publishes accompanying datasets in dedicated data repositories^5^.

In this paper we present the Text Reuse at Scale interface for the visualization-aided discovery which will complement the *impresso* application. The new interface facilitates “scalable reading” of text reuse in historical media for historians and other scholars in the humanities. Scalable reading combines close and distant reading. In the case of newspapers, close reading corresponds to either the inspection of individual text reuse clusters and the passages they contain or the study of the articles to which they belong. Distant reading refers to the distributions of text reuse measures, metadata, and semantic enrichments. We describe generic (media) historical research objectives and identify a list of generic tasks for the exploration of text reuse data.

The interface design and tasks were partly informed by the outcomes of a two-day workshop organized by the *impresso* team, which brought together a group of 10 researchers (from inside and outside the *impresso* project), including professionals from various disciplines such as design, natural language processing, data science and (media) history. The workshop produced a list of historical research objectives (also in the light of previous work), associated tasks and three interface mockups.

The structure of this paper roughly follows our interface creation process. Section 2 positions our work in relation to the state of the art: it introduces the methods and tools for TRD, discusses recent advances and remaining challenges for detecting and exploring text reuse in historical newspapers, and concludes with a brief presentation of the *impresso* text reuse data. Turning to historical research interests in text reuse data, Section 3 focuses on five high-level research objectives in (media) history and associated 11 generic tasks that we identified. Next, Section 4 presents the prototype interface in relation to case studies illustrating specific tasks, and Section 5 reports on the results of an evaluation undertaken by 13 users. Finally, Section 6 closes the paper with an outlook on future work.

## 2. State of the art: text reuse detection in historical texts

This section situates our work in the current state of the art in text reuse detection and usage for humanities research. We begin with an overview of tools and methods, continue with current directions in the visualization-aided exploration of text reuse data, and conclude with a description of TRD in the context of the *impresso* project.

### 2.1. What is text reuse and how is it detected?

Methods for TRD are shaped by the disciplines in which they emerged. Since text reuse in literary texts is often more subtle than the mere repetition of words (e.g., in the case of paraphrase, allusion, translation, or parody), researchers strive to go beyond lexical similarities in order to capture affinities in syntax, content, or metrical structure (Büchler et al., 2014; Scheirer et al., 2016; Moritz and Steding, 2018). In the design of TRACER^6^, Büchler et al. (2014) have addressed this subtlety of text reuse in literary texts by giving users access to a wide array of Information Retrieval (IR) algorithms, as well as direct access to the tool's output at each step of the processing chain. More recent studies have investigated the usefulness of sentence and word embeddings, especially with respect to detecting these more allusive forms of text reuse (Manjavacas et al., 2019; Liebl and Burghardt, 2020), finding that they do not bring substantial advantages over traditional IR techniques.

On the other hand, the challenges of detecting text reuse in the newspapers domain are quite different. The substantial amount of OCR noise present in digitized newspapers asks for fuzzy methods that are resilient to differences between two or more copies of the same textual content. Moreover, the scale of materials—with corpora that can be several orders of magnitude bigger than those in the literary domain—led to the development of efficient and scalable methods. As a matter of fact, methods that were developed for TRD in the newspapers domain had to deal with both challenges, namely OCR noise and scalability. Vesanto et al. (2017) adapted the Basic Local Alignment Search Tool (BLAST) algorithm, originally developed for the alignment of biomedical sequences, to the task of character alignment.^7^ An alternative approach to TRD consists in performing alignments between documents at the level of longer sequences of words, a.k.a. n-grams, instead of individual characters. This was the approach followed by Smith et al. (2015) whose TRD algorithm, implemented in the tool passim^8^, uses n-gram-based filtering to reduce the number of text passage pairs to compare—thus achieving scalability—and combines it with local and global alignment algorithms to handle gaps and variants in longer sequences of aligned texts.

### 2.2. Interactive visualizations of text reuse

Text reuse instances can be visualized, analyzed, and explored at various levels:

- *Corpus-level* analysis considers all text reuse instances within a corpus; the size and composition of corpora vary; user-defined collections can also be considered as corpora in their own right. Scalability is a typical challenge for visualizations at this level of analysis.
- *Document-level* analysis considers all text reuse instances within a single document or across sets of documents; compared to the corpus-level, this level of analysis is more meaningful for longer documents such as entire books or book chapters, but it can be applied as well to shorter documents such as journal articles. When applied across documents, this approach provides insights into the genealogy of texts (multiple versions of the same book, different books that have borrowed from one another).
- *Cluster-level* analysis considers one single instance of text reuse, with a specific focus on higher-level patterns (e.g., diachronic development of a cluster as a proxy for information spreading).
- *Passage-level* analysis considers a single instance of text reuse but focuses on existing differences between (pairs of) witnesses (i.e., text passages that are deemed to contain the same text despite some variations). The possibility of inspecting text reuse witnesses in their original broader context (i.e., the position of a reused passage within the book or newspaper page) is an important aspect of the contextualization of the reused text.

Generally, distant reading approaches tend to privilege analysis of corpus-level and document-level text reuse, while the close reading approach is more concerned with cluster-level and passage-level reuses. Existing interactive visualizations of text reuse tend to support multiple levels of analysis at once and often allow users to seamlessly move between levels. Visualization techniques for cluster- and passage-level text reuse resemble those used to represent text alignment in other scenarios, e.g., translation alignment, collation of sources, etc. (Yousef and Janicke, 2021).

The interface developed for the *Graph–Text reuse in rare books*^9^ project constitutes a compelling example of interfaces supporting multiple levels of exploration. It was developed to enable the exploration of text reuse passages extracted from a corpus of 1,300 OCRed rare books. Firstly, corpus-level text reuse is represented as a graph where two nodes (books) are connected when they contain reused passages, with the additional possibility of ordering the graph by time (of publication). Secondly, a static alluvial diagram allows readers to inspect more closely document-level reuse between pairs of books; this is especially useful to understand flows of reused text across books. Lastly, a facsimile side-by-side view of pairs of books permits to focus on passage-level reuse. This viewer is not aimed at highlighting differences between reuse passages, but rather at displaying them in their original context (especially meaningful in the case of rare books).

Graph visualization of text reuse at corpus level has also been used in the context of the Viral Texts project, which studied virality in newspapers during the interwar period. An interactive network visualization^10^ developed by the project provides a bird's eye-view of millions of text reuse passages, distilled into a graph that shows how newspapers formed a network of reprints and content reuse. Node size and color are used to express node centrality and grouping into community clusters, respectively, while the thickness of edges connecting nodes indicates the number of shared reprints. In addition to network visualization, geographical maps were used to support cluster-level analysis, as they allow to visualize at a glance the geographical distribution of reprints of a given text (Cordell, 2015).

Finally, visualizations of text reuse for the study of reception—be it literary or scholarly–privilege the corpus-level analysis of text reuse data and tend to present them in some aggregated form. In fact, what matters for the study of reception is how repetitions (quotations) are distributed, rather than the fine-grained differences between them. Examples of text reuse visualizations geared toward the study of reception are *Cited Loci of the Aeneid* (scholarly reception of Vergil's *Aeneid*) (Romanello and Snyder, 2017) and the *Reception reader* which focusses on Victorian literature (Rosson et al., 2023).

### 2.3. Text reuse detection in the *impresso* project

**Figure 1:**
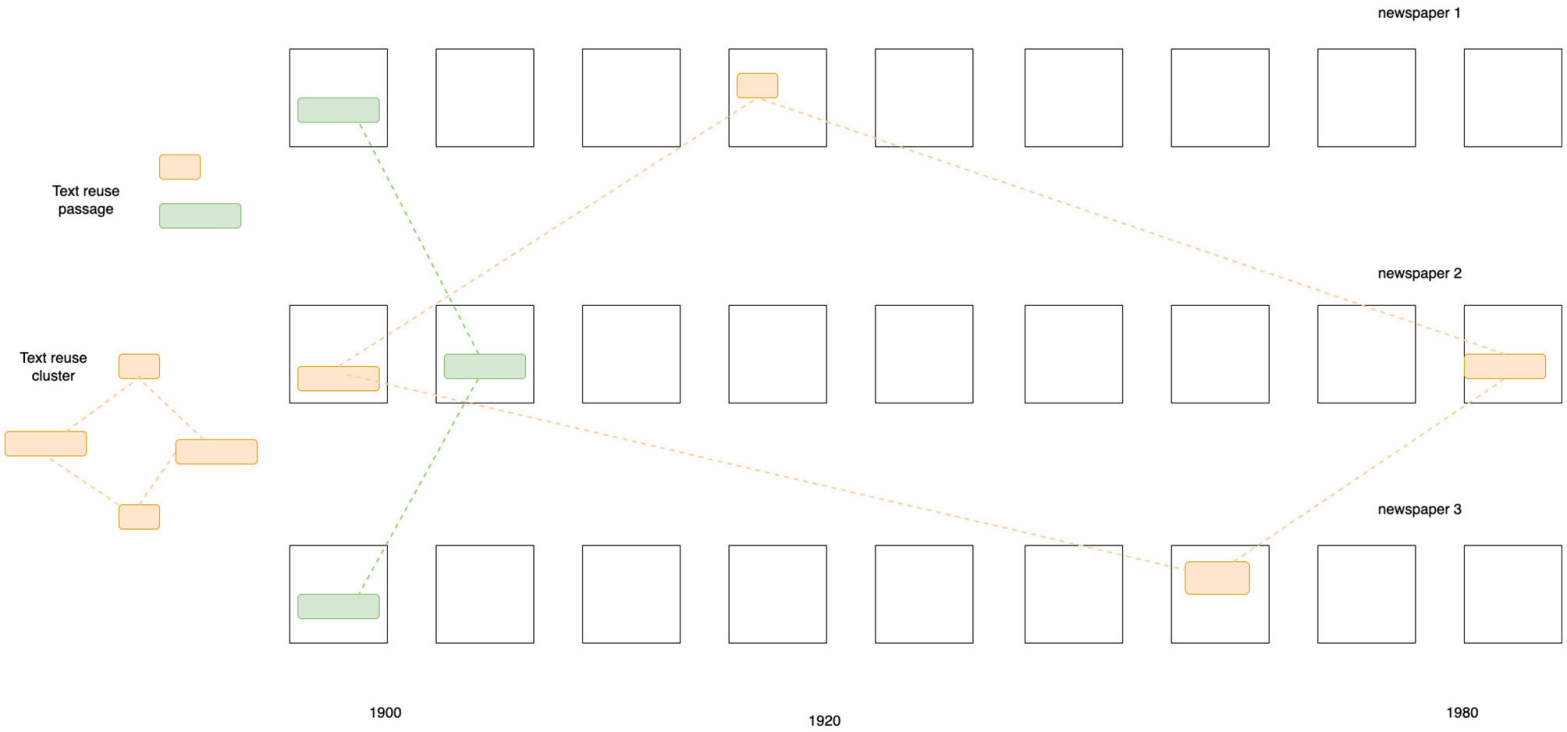
Schematic view of text reuse clusters and passages extracted from a newspaper corpus.

We used the open-source software Passim (Smith et al., 2015) to detect text reuse within the *impresso* corpus. Passim outputs clusters, groups of newspaper passages (or witnesses), from different newspapers, that share a common text span—the reused passage—of varying length (see Figure 1). The reason for choosing Passim over existing alternatives^11^ was its ability to scale up, guaranteed by the software's parallel computing architecture. Preliminary tests on the *impresso* corpus showed that Passim's fuzzy alignment algorithm was able to detect reuse despite the presence of (moderate) OCR noise.^12^

#### 2.3.1. Text reuse detection and processing

As a pre-processing step, we ran Passim in boilerplate detection mode; this allowed us to identify—and later filter out—boilerplate content present in our corpus, i.e., portions of text that are repeated within the same newspaper in a 1-month window (as opposed to reuse across different newspapers). All content items where boilerplate text was detected were filtered out from Passim's input. This pre-processing step allowed for reducing the final number of detected text reuse clusters by removing some noise from the input data. After filtering for boilerplate text, we extracted 6,177,815 text reuse clusters, for a total of 16,099,821 reused passages. The reused passages are contained in 8,111,123 content items, meaning that roughly 17% of all content items in the corpus (*n* = 47,798,468) are part of at least one text reuse cluster.

We then post-processed Passim's output to enrich the detected clusters with the following information (see also Table 1):

- *Cluster size*: the number of passages contained in a cluster;
- *Lexical overlap*: the percentage of unique tokens that all passages in a cluster have in common (all text is lowercased and punctuation is stripped);
- *Time span*: the time window covered by documents in the cluster, expressed in number of days. It is computed as the difference between the publication date of the oldest and the most recent content item in the cluster.

**Table 1:** Text reuse measures and their representation in the interface.

**Measure**	**Description**	**Implementation in interface prototype**
Passages per year	Number of passages counted in a given year.	Line chart which displays the count of passages per year for a given query or filter operation. This gives a first indication, during which years text reuse occurred more commonly. Time sliders and precise date entry allow users to filter for exact date ranges to inspect.
Cluster size	The number of passages contained in a cluster.	Histogram which shows the distribution of text reuse cluster sizes and indicates the highest score. The histogram groups clusters of size n and displays their sum. This gives a first indication of averages as well as outliers. Sliders can be used to specify a cluster size range of interest. Filtering by cluster size allows to exclude or explicitly focus on outliers but different cluster sizes may also correspond to different types of content.
Lexical overlap	The percentage of unique tokens that all passages in a cluster have in common. All text was lowercased and punctuation was stripped.	Histogram which shows the distribution of lexical overlap in percent and indicates the largest number of clusters for a given score. Extremely low lexical overlap decreases the chance to discover meaningful text reuse whilst extremely high overlap will only reveal near-copies of content and may be too restrictive for some purposes.
Time span	The time window covered by documents in the cluster, measured in number of days.	Histogram which shows the gap between the earliest publication date of an article in a text reuse cluster and the latest measured in days and indicates the largest number of passages for a given score. This is an efficient approach to discover or filter for instances of slow, mid-range and rapid text reuse. The histogram groups clusters by the number of days in between publication dates and displays their sum.
Text reuse clusters	Clusters store text segments (or passages) that are reused in different units of a corpus.	List of text reuse clusters which match a given query, sorted by number of passages. Each cluster is characterized with basic information (passages count, lexical overlap, time periods and years covered) as well as a snippet preview of the passage. Clusters are sorted by the number of matching passages. Clusters can be selected manually for further inspection in the Text Reuse app or in other *impresso* components such as Search.

#### 2.3.2. Integration of text reuse data in *impresso*

**Figure 2:**
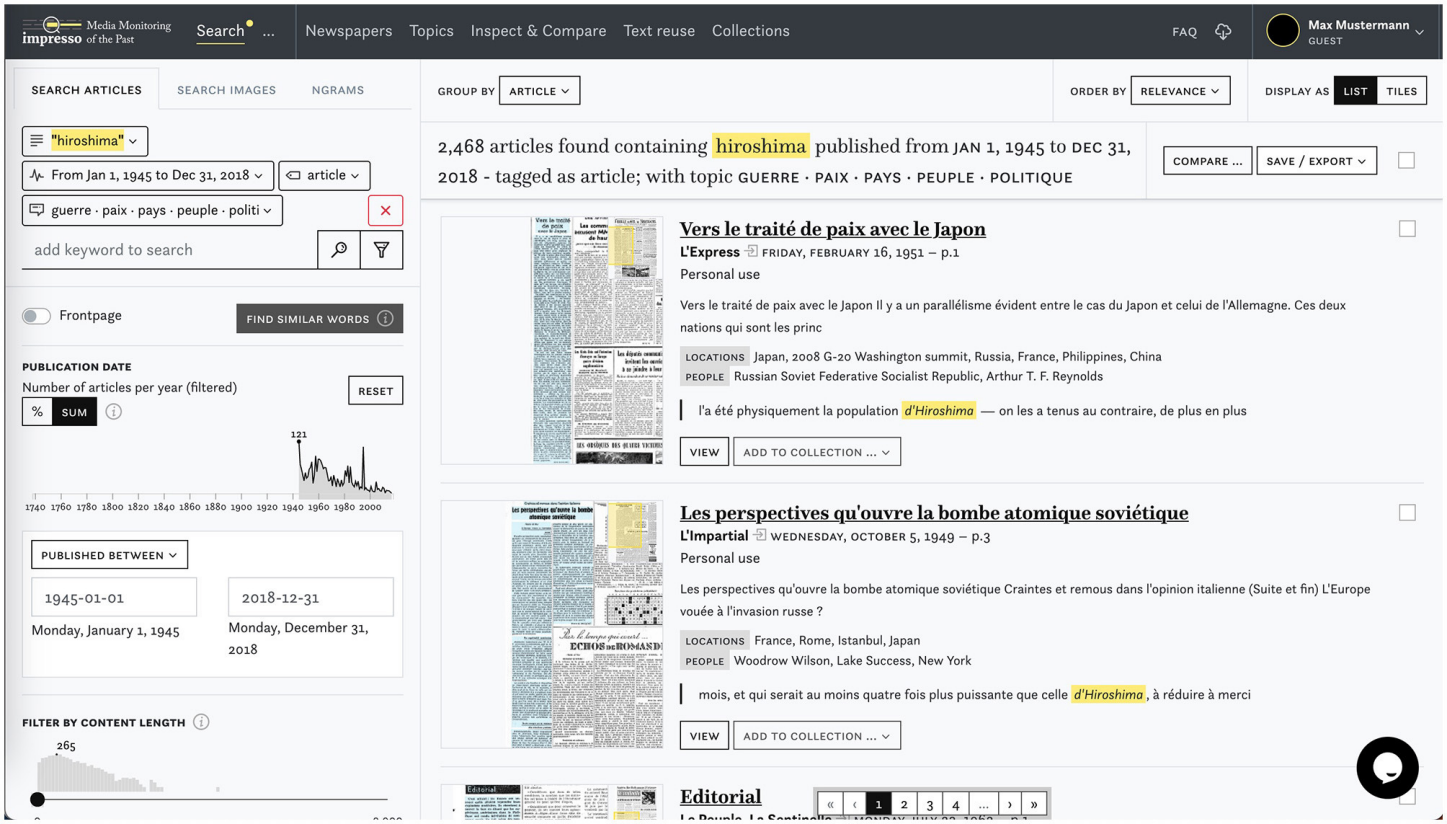
Screenshot of the *impresso* application for the exploration of semantically enriched historical newspapers.

**Figure 3:**
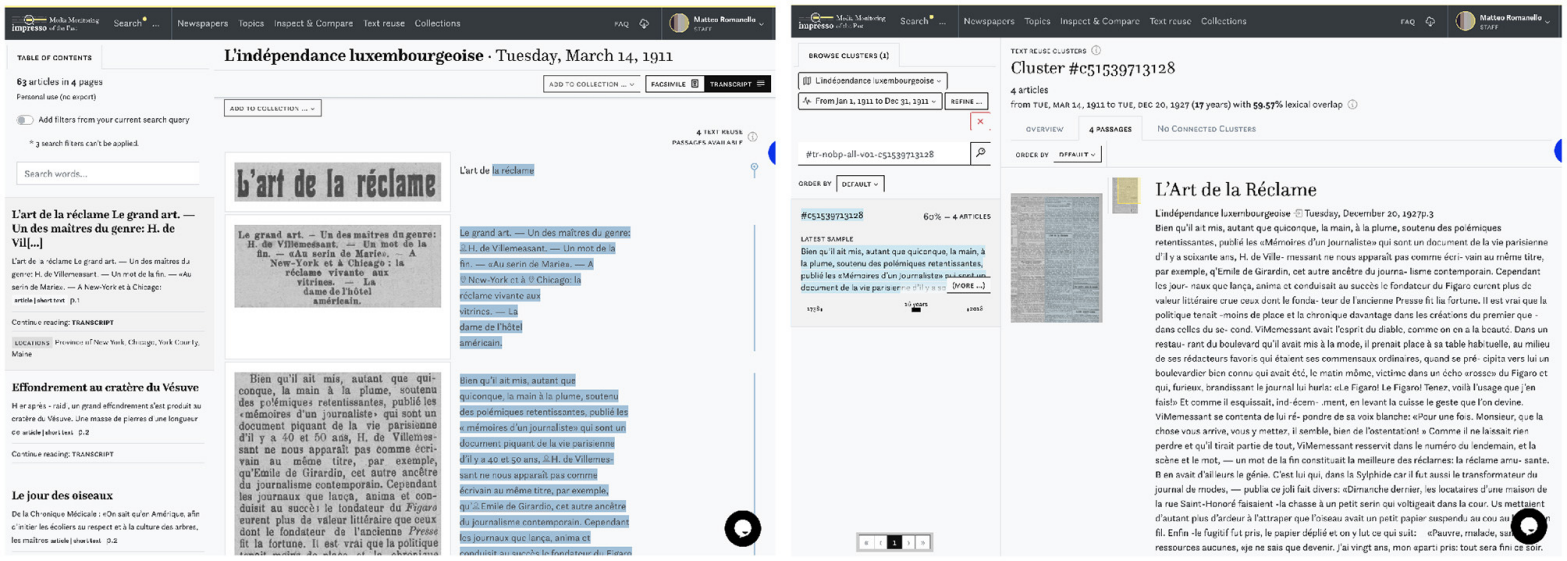
Display of text reuse in the *impresso* application's article reading view **(left)** and first version of the text reuse explorer **(right)**.

Text reuse data are already integrated and displayed in two main parts of the current *impresso* application (Figure 2). First, in the article reading view, colored highlights indicate to the reader which parts of an article are reused elsewhere in the corpus. Second, in a text reuse explorer that precedes the prototype interface we discuss here (Figure 3). This first version already allows users to browse, search or filter text reuse clusters by any of the characteristics computed in the post-processing step, such as cluster size (i.e., number of passages contained), lexical overlap or time span covered. Most importantly, users can filter clusters to keep only those found in one of their collections. This functionality allows to *reveal* the presence of text reuse within a carefully selected and possibly manually curated subset of corpus.

One of the main difficulties we faced in integrating text reuse into the *impresso* application was the scale of data, and more specifically how to enable an effective exploration of millions of detected clusters. Our approach to this problem consisted in providing users with as many filters as possible, as a powerful way of sifting through the large number of clusters extracted by Passim. One example is the long-term reuse of newspaper contents (Salmi et al., 2019), i.e., articles that are reprinted over and over in a relatively long period of time: Users can refine their query by setting a filter on the time span of the cluster, so that only clusters consisting of articles covering a time span of, e.g., 10 years are retained. This first version mainly supports cluster- and document-level research with a basic set of search options and filters without distant reading perspectives.

The development of the new version was motivated both by the opportunity to fully leverage the available enrichments and by the prospect of supporting additional use cases, including passage- and corpus-level research. To this end, we integrated text reuse and semantic enrichment data, i.e., named entities, topics, and content item types (where available), and aligned them with text reuse passages and clusters.

## 3. Historical research and text reuse

After reviewing the state of the art in TRD for historical texts, this section discusses the motivations and needs of historians interested in working with newspaper collections.

Past and present media have been, and still are entangled in complex communication networks, manifested in the form of interactions between different stakeholders such as journalists and press agencies. Connectivity in such networks was influenced by various factors, including geography, politics, technology, communication infrastructures, languages, and commercial interests. By studying how texts circulated, historians can reconstruct the emergence and dissolution of links between stakeholders across time and space. Studies of copy-paste journalism, plagiarism, paraphrasing, literary and scholarly citation, the dissemination of specific discourses, and similar phenomena therefore all stand to benefit from TRD.

TRD reveals many different types of text reuse such as jokes, adverts, boilerplates, speeches, or religious texts, but also short stories and reprints of book segments. Each of them is tied to a different logic and motivation and enables researchers to study different aspects of past media. We identify five high-level historical research objectives in the study of historical media and subsequently derive 11 common tasks from these objectives.

### 3.1. High-level historical research objectives

Within historical research objectives, we distinguish between media-centric and content-centric perspectives. Media-centric perspectives seek to understand the functioning and evolution of the press as a system of information production and dissemination. Content-centric perspectives use historical media to reconstruct public attitudes and focus on the representation of past discourses.

#### 3.1.1. (Trans-) national media ecosystems

With the increasing availability of digitized newspaper collections, media historians have begun to broaden the scope of their analyses: Attention has shifted from the in-depth reconstruction of the history of individual titles to a broader approach that embeds them in a transnational media ecosystem and emphasizes the critical role of such connections in the creation and dissemination of information. Current research seeks to understand the functioning of this ecosystem and the agents which shaped it. This includes questions about the underlying ideological, commercial, and financial structures on which historical media ecosystems were based. Previous research, for example, has shown the importance of telegraph lines and railways in the spread of information within the United States (Smith et al., 2013) and pointed to individual cities as information dissemination hubs (Cordell, 2015; Salmi et al., 2020). Other work has studied multilingual information flows from a transnational perspective to examine the connections, gaps and silences in the system, and the press as a site of manipulation (Keck et al., 2022; Paasikivi et al., 2022; Paju et al., 2023).

The increasing availability of text reuse data for different countries will further advance systematic analyses of transnational (re-) printing dynamics. Of particular interest are e.g., internationally operating press agencies with their ability to disseminate content across borders and languages nearly simultaneously.^13^ A transnational perspective reveals the ways in which news is altered and contextualized as it travels. Scrutinizing the reproductions of text by examining additions and deletions as traces of adaption helps us understand what was considered common knowledge in one (national) context but not in another. It foregrounds how perceptions and descriptions are adapted to new audiences.

#### 3.1.2. Newspaper content as *bricolage*

From a linguistic perspective, newspaper discourse is not necessarily original or innovative. Many genres such as weather forecasts or sports reporting operate within constraints and happen to be almost formulaic. Walma (2015) and Thèrenty and Venayre (2021) paid special attention to the relations between these genres: how did content travel between them? In general, articles emerge through a process of creative re-use and re-appropriation. Whole fragments, sentences and quotations are often transferred to novel contexts. In this sense, newspaper content emerges through a process of what could be called *bricolage*, in which content is soldered together from existing fragments and textual patterns. In other words, newspapers content is often harvested from a wide range of available textual material.

This research objective investigates text reuse through the angle of compilation and content production. Text reuse measures can be used to encode textual relations and connections, and thereby enable researchers to critically disentangle the genesis of newspaper content. Moreover, the concept of *bricolage* opens up a graded, more nuanced approach, to the study of text reuse: it foregrounds how the creation of news content emerges in a complex process of transformation, compilation and innovation. Newspaper titles operate in a media ecosystem compiling and recreating content harvested from the “grid” (press agencies, or newspapers) and merging it with self-generated content (ads, journalistic work, external contributors etc.).

#### 3.1.3. Historicising virality

Virality is more commonly understood as a phenomenon of the internet era and is associated with three characteristics: High speed, high volume and the ability to adapt and spread quickly. Paju et al. (2022) have used text reuse data in an attempt to measure and compare different degrees of virality for content that was republished within days or weeks. They define a virality score based on the number of titles within a cluster, the number of unique printing locations, and the distance in days between the first and last passage publication date. They show that different types of content qualify for different types of repetition: An advertisement for Finnish cigarettes in 1916 constituted the most viral content in their corpus while institutional announcements, literary, and religious texts often fall into the mid-range virality category.

Such measures may yield additional insights into the functioning of historical media ecosystems, e.g., by revealing which types of texts circulated more efficiently than others within and beyond national boundaries, and who was responsible for their creation and dissemination. Virality also offers insights into how transport infrastructures and geography shaped information dissemination, or how other factors such as religious and political affiliations influenced the reception or rejection of content.

#### 3.1.4. Tracing historical events

The press is a system of knowledge production and representation that not only presents events to the public, but also situates them within a specific political, economic, social, and cultural context. This influences the perception of historical events by the public. A comparative approach to event coverage therefore allows us to reconstruct the political, social, and cultural identities of individual newspaper titles and how they evolved over time. We identify two strategies to trace the coverage of historical events via text reuse. The first is bottom-up and concentrates on individual, known events and the question of whether or not they were picked up by the press and if so, how. For example, Oiva et al. (2020) studied how news of the assassination of Nikolay Bobrikov, the Governor-General of Finland in 1904, traveled in waves across historical communication infrastructures. The second strategy is top-down and focuses on the types of events that successfully spread across media ecosystems. For example, Keck et al. (2022) used TRD across newspaper collections from the United States, Britain, Germany, Austria and Finland to identify global media events. Through this approach, they discovered a substantial number of articles that circulated during Hungarian revolutionary Lajos Kossuth's tour of America to seek US financial support for another revolution in Europe. His arrival in New York in December 1851 and his subsequent travels to Washington, DC triggered a proliferation of coverage and reprinted texts. Comparing text reuse across national and linguistic borders highlights the specific patterns and complexities of transatlantic news circulation, including pathways, reach, temporality, vagaries, and gaps. While this work illustrates the usefulness of TRD, it also highlights the benefits of international cooperation when working with multilingual datasets.

#### 3.1.5. Capturing historical Zeitgeist

To some degree, historical media record the attitudes, norms, beliefs, moods and feelings of past generations, and can thus serve as a proxy for the study of “Zeitgeist.” This concept alludes to notions of similarity and parallel evolution, and explains how texts which were produced independently come to share certain characteristics. Because of its fuzziness, Zeitgeist marks the borderline of what text reuse can capture. Zeitgeist manifests itself in various forms, such as mental maps that informed the editing of texts, adverts for (cultural) products, or the mere existence of coverage of cultural practices. These manifestations are created using persistent and implicit templates that change their content over time—an example would be dance fads such as Polka or Macarena, which are dominant at one point but then slowly fade away. Related work looks at conceptual change over time (Verheul et al., 2022) or the cultural impact of Cholera epidemics (Paasikivi et al., 2022).

### 3.2. Tasks for the exploration of text reuse in historical newspapers

This section operationalizes the high-level objectives by formulating concrete user tasks. The tasks are not directly linked to objectives, but provide building blocks that compose workflows for the exploration of text reuse data. Table 2 relates these tasks to analytical levels described in Section 2 and also mentions the degree of support provided by the interface.

**Table 2:** List of tasks and current degree of support by the Text Reuse at Scale interface.

**Task**	**Title**	**Level**	**Support**
1	Obtain an overview of text reuse in a corpus, collection or query	Corpus	Yes
2	Obtain an overview of a single cluster	Cluster	Yes
3	Compare passages	Passage	Yes
4	Compare clusters	Cluster	Yes
5	Identify different types of text reuse	Corpus	Yes
6	Generate research corpora based on text reuse clusters	Corpus	Yes
7	Identify connections	Corpus	Partial
8	Detect and trace virality	Corpus	No
9	Search for passages	Passage	No
10	De-duplicate content	Corpus	No
11	Export of text reuse data	All	Planned

**Task 1: Obtain an overview of text reuse at the corpus, collection or query level**. Before analysis, users need to determine whether or not a given dataset, such as a corpus, a corpus subset or a query result, contains instances of text reuse. Metadata plays an essential role in contextualizing the presence of text reuse data. In the case of the *impresso* data, this includes publication dates, newspaper titles, country of publication, content types, languages, topics, and named entities. It also consists of text reuse specific measures such as lexical overlap, time span between publication dates, cluster size and number of passages.

Computing measures of spread offers additional insights into the distribution of text reuse data at different levels of granularity. This includes finding the largest/smallest clusters, clusters with the highest/lowest lexical overlap, the earliest/latest cluster, or clusters with the longest time span between publication dates.

**Task 2: Obtain an overview of a single cluster**. This task is similar to Task 1 but focuses on the properties of a single cluster: the number of passages, their content, the lexical overlap between them, the time span and rhythm between publication dates, and the distribution of metadata.

**Task 3: Compare passages**. This task compares two or more passages and reveals textual differences and similarities through parallel reading. A common use case is the study of editorial changes in news agency dispatches. Comparisons can reveal adaptations to suit the political preferences of a newspaper's audience, additional explanations and clarifications to address varying knowledge horizons, but also unintended differences such as text degeneration caused by OCR errors.

**Task 4: Compare clusters**. Comparison is a powerful analytical instrument. This task compares sets of clusters based on the distribution of (a) text reuse measures and (b) metadata and semantic enrichments such as topics or named entities.

**Task 5: Identify different types of text reuse**. TRD captures many different types of reused texts that need to be distinguished from each other. In combination, semantic enrichments, TRD data and their temporal distribution (see Table 3) provide powerful means to identify different types of text reuse. For example, older articles that are reprinted after years, or advertisements that were widely published in parallel, tend to contain named entities and topics that are typically associated with particular types of media content.

**Table 3:** Types of temporalities in text reuse in historical newspapers.

**Type**	**Description**	**Measures**	**Examples**
Duration	The time period which is covered by a cluster ranging from the earliest to the latest publication date of individual passages.	Publication date	Paju et al.'s notions of fast and slow text reuse fall into this category.
Virality	The speed (measured in days) and breadth of text reuse passages spreading within a corpus. Speed corresponds to time passed (e.g., days) whereas breadth corresponds to the number of publications which contain a passage at a given point in time.	Publication date, number of publications	News of the sinking of the Titanic or the destruction of the Hindenburg Zeppelin traveled around the world within days or weeks.
Rhythm	Pattern with which text reuse passages appear over time.	Distance between publication dates	Reprints of articles on the occasion of their anniversary, e.g., on the occasion of the bombing of Hiroshima.

**Task 6: Generate research corpora based on text reuse clusters**. This task supports the fine-grained selection of content and the creation of meaningful subsets of text reuse clusters and their associated passages based on the aforementioned measures, enrichments and metadata.

**Task 7: Identify connections**. This group of tasks concentrates on the relational structure of media ecosystems. Examples include the (unwanted) reprinting of articles by different newspapers, the exchange of content between news agencies, or regular co-publication agreements between newspaper titles.

**Task 8: Detect and trace virality**. This task corresponds to the pioneering work of Salmi et al. (2020) and measures the efficiency and speed by which content spreads across newspapers.

**Task 9: Search for passages**. This task describes a search scenario in which a seed text is used as a query and compared to known text reuse passages. An example usage would be the upload of a speech to determine whether parts of it were ever published in a given corpus.

**Task 10: De-duplicate content**. This processing step removes duplicate texts, e.g., to avoid over-representations of highly circulated texts or recurrent elements such as adverts or boilerplate.

**Task 11: Data export**. Data export allows further processing outside the constraints of an application. This would allow network or geo-spatial analyses or further processing.

## 4. The *impresso* Text Reuse at Scale interface

### 4.1. Main interface components

**Figure 4:**
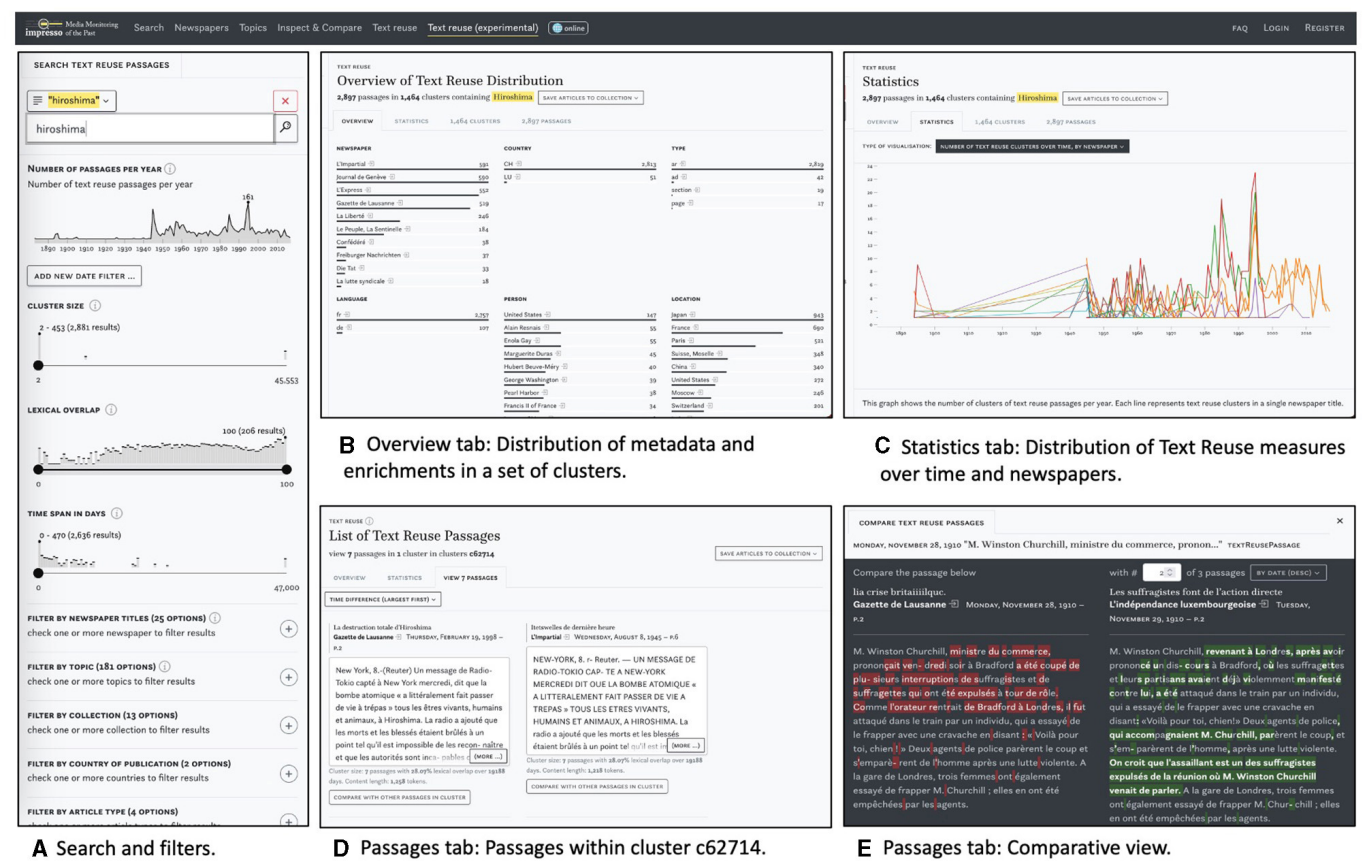
Main components of the text reuse interface: search and filter pane **(A)** and the overview **(B)**, statistics **(C)**, and passages tabs **(D, E)**.

The *impresso* Text Reuse at Scale interface consists of a search and filter pane on the left and three tabs in the center (see Figure 4). This section introduces these components and provides examples to illustrate their usage.

#### 4.1.1. Search and filter pane

Figure 4A shows the search and filter pane. Users can compile queries using the versatile *impresso* Search component together with a variety of filters. These filters include newspaper metadata, user-generated article collections, text reuse clusters, and semantic enrichments (topics, language, content type, and named entities). In addition, the component allows filtering based on the number of passages over time, lexical overlap within clusters, cluster size and time span (for details see Table 1).

**Figure 5:**
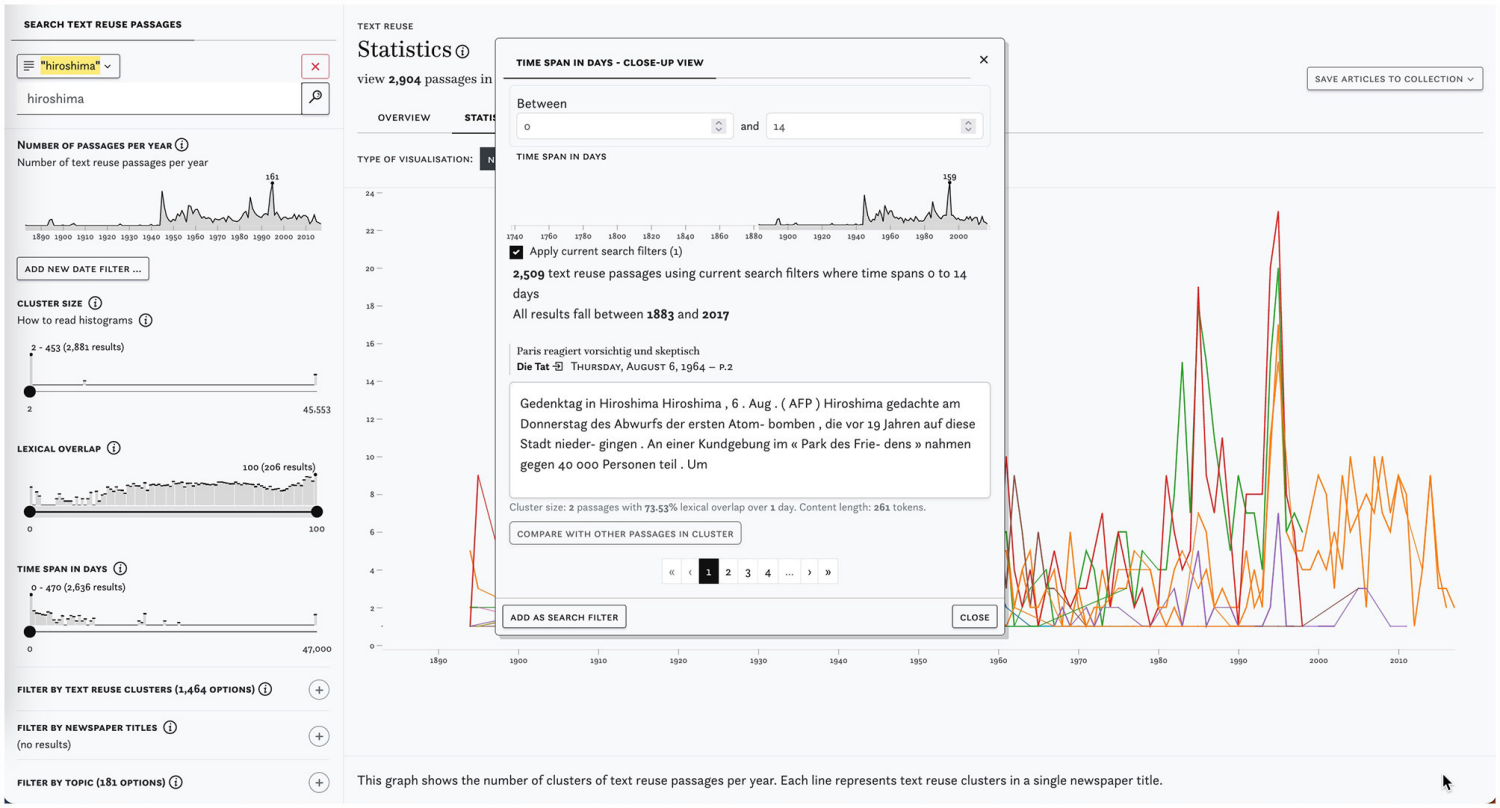
Screenshot of the search and filter pane with a keyword search for *hiroshima* **(left)** and the close-up view with a time span filter for 0 to 14 days **(center)**.

Complementary modal dialogues as shown in Figure 5 (center) serve as a bridge between distant and close reading. They reveal, for example, notable peaks in the distribution of lexical overlaps, cluster sizes and time spans between publication dates and allow users to quickly browse corresponding passages.

Taken together, these search and filtering capabilities offer a versatile framework for querying text reuse data. As a first illustration, different types of text reuse can be retrieved by filtering based on the time span between passage publication dates as described in Task 5–Types. Paju et al. (2023) point to the different speeds at which text reuse occurs, distinguishing between rapid (within 1 year) and mid-range (up to 50 years) cycles. Anecdotal evidence suggests that slow text reuse (up to 140 years) is typically tied to conscious re-prints of archived materials. Following Paju et al.'s classification of text reuse speeds, we find 5,546,859 clusters that qualify as rapid (0–365 days), 557,555 clusters that qualify as mid-range (1–50 years), and 21,799 clusters that qualify as slow (50–200 years).

As another illustration, let us consider the press coverage of the US attack on the Japanese cities of Hiroshima and Nagasaki on 6 August 1945. We begin with a basic keyword query for *hiroshima* which yields 2,897 passages in 1,465 clusters. We note clusters with very short time spans (0–2) concentrated in 1945 and 1995. Upon closer inspection, cluster c466008 stands out: it includes 9 passages from articles that were republished surrounding the anniversaries of the attacks, making it an example of cyclical text reuse. The Swiss newspaper *L'Impartial* published them with minor changes irregularly between 2007 and 2015 in commemoration; in 2009 and 2012 the article was also republished by *L'Express*.

**Figure 6:**
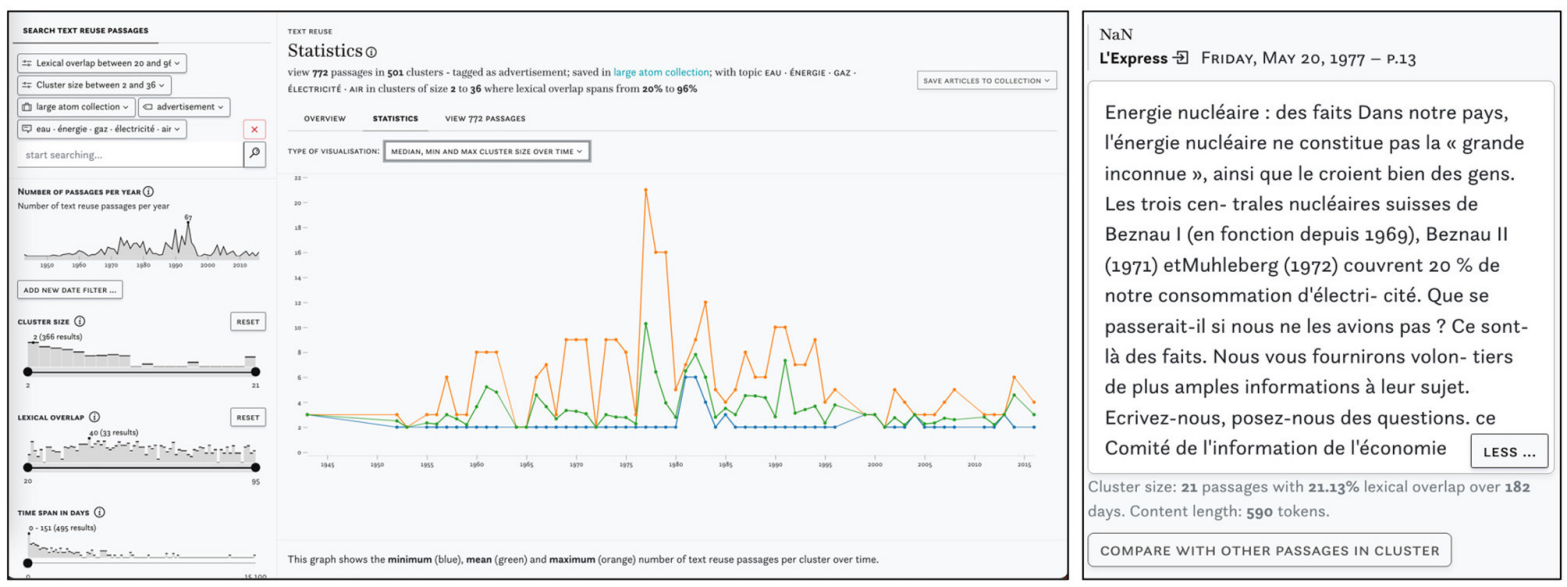
Example of a complex query using multiple semantic enrichments and *impresso*'s collections. Distribution of clusters on the left and passage from the largest cluster in 1977 on the left.

Finally, Figure 6 shows a query for text reuse in adverts that are part of a large collection of articles about nuclear power and linked to the topic *eau énergie gaz électricité air*. The peak in 1977 points to cluster c276252 which comprises 21 adverts in favor of nuclear power, published in parallel in several Swiss newspapers.

#### 4.1.2. Overview tab

Figure 4B displays the *Overview* tab which was inspired by Task 1–Overview. It shows the distribution of semantic enrichments and metadata relative to a search or filtering operation. In this case, it displays the results for the preceding keyword query for the string *hiroshima*. Enrichments are grouped by type and represented using small multiples of bar charts.

In this instance, we learn that the vast majority of text reuse passages that contain *hiroshima* are linked to content published in French in Switzerland. Content published in German in Luxembourg remains the exception. A closer look at the newspaper titles suggests that roughly 80% of these passages appear in just four newspapers. Unsurprisingly, the most prominent topics are associated with war, nuclear technologies, and aviation.

#### 4.1.3. Statistics tab

**Figure 7:**
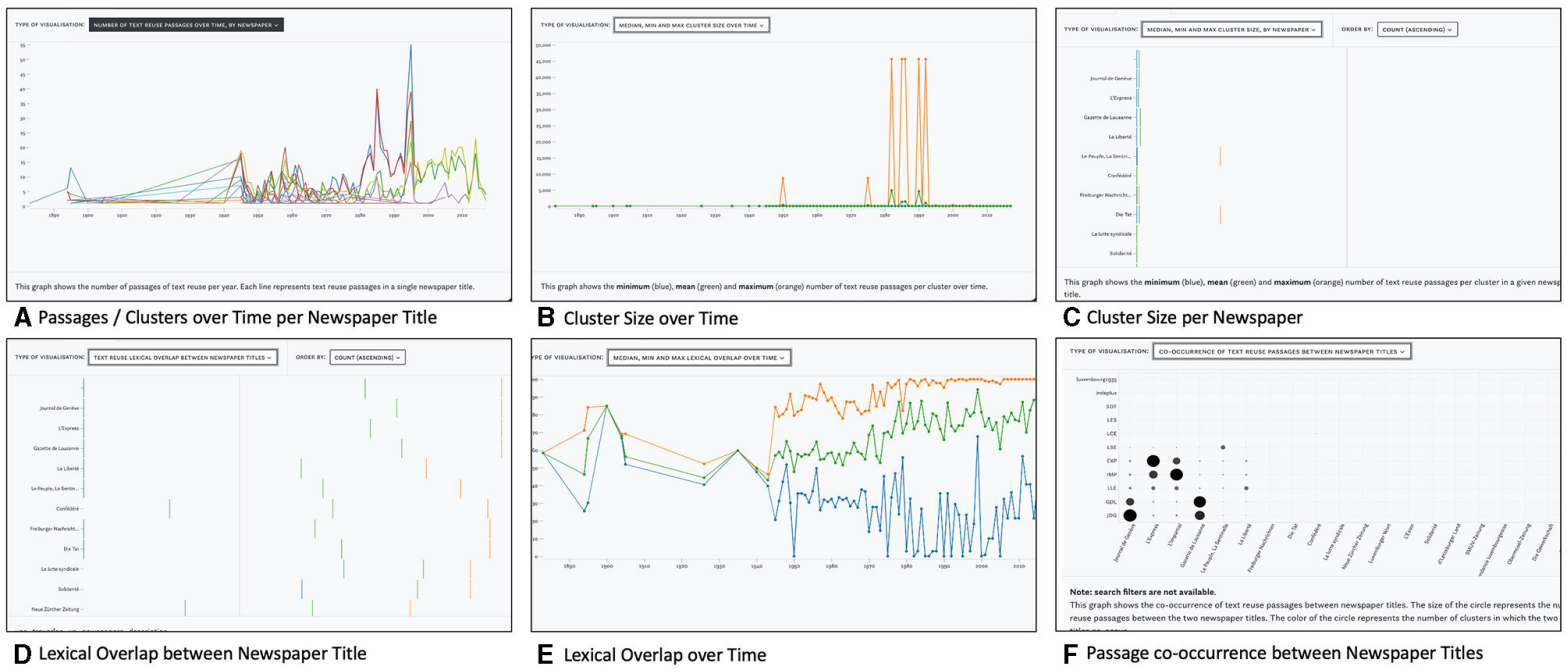
Views in the Statistics tab with distributions of text reuse measures across time and newspaper titles. Includes a graph to display passages or cluster counts over time per newspaper title **(A)**, cluster size over time **(B)**, cluster size per newspaper **(C)**, lexical overlap between newspaper titles **(D)**, lexical overlap over time **(E)**, and Passage co-occurrence between Newspaper titles **(F)**.

The second tab, *Statistics*, visualizes the distribution of text reuse measures in relation to queries and provides a distant reading perspective. A drop-down menu offers access to five views: four line charts and a matrix visualization, which are shown in Figure 7 and discussed in more detail below.

Figure 7A displays the **passage count over time by newspaper title**. This view reveals periods of heightened or reduced text reuse activity for one or more newspaper titles. A complementary view represents the number of clusters over time (not shown).

**Figure 8:**
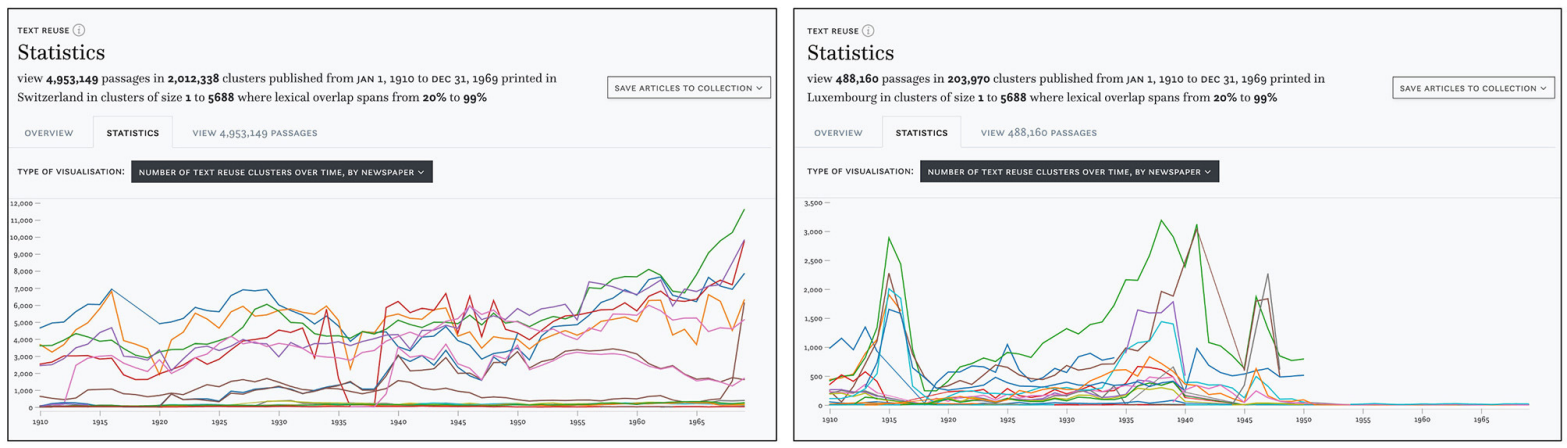
Number of clusters detected in Swiss **(left)** and Luxembourgish **(right)** newspapers 1910–1970.

As an example, we will compare the distribution of text reuse in Swiss and Luxembourgish newspapers. In the search and filter panel, we set the time delta filter to a range between 0 and 100 days. Lexical overlap is set to a moderately high range of 20–99%, which should also retrieve reused text segments of smaller size embedded in a larger text. Finally, we use the cluster size filter to exclude a disproportionately large cluster of 45,000 passages. This results in approximately 5.5 million passages for our time period. Looking at the distribution of cluster sizes in Switzerland, Figure 8 (left) suggests some variation between titles, but otherwise no change between the pre- and post-war period. In contrast, the Luxembourgish press Figure 8 (right) exhibits a growing number of passages since the 1930s and clear peaks in 1915 and during the Second World War, followed by a stark decline after 1950 which can be explained by the limited availability of Luxembourgish content for this time period in our corpus.

The **minimum, mean, and maximum cluster sizes over time** are shown in Figure 7B. Overall, the number of text reuse clusters and passages rises constantly over time, parallel to the number of available content in the *impresso* corpus. For another example, we make use of the *impresso* Collections feature which stores sets of articles based on either manual selection or queries (see Task 6–Research corpora). Collections can also be created based on text reuse clusters—albeit with the caveat that it saves both passages and entire articles.

Figure 7C captures the **minimum, mean, and maximum cluster sizes per newspaper**. This view depicts the distribution of cluster sizes across titles and shows which newspaper produced the smallest (or largest) clusters. In this case, both the newspapers *Le Peuple, La Sentinelle* and *Die Tat* stand out with above average maximum cluster sizes (orange).

**Lexical overlap between newspaper titles** is shown in Figure 7D while Figure 7F uses a matrix view to highlight **co-occurring text reuse clusters between newspaper titles**. Both views reveal particularly high lexical overlaps and a large number of shared passages for *Journal de Genève* and *Gazette de Lausanne*. These findings confirm our prior understanding of the frequent co-publication patterns among these titles.

Finally, the distributions of **lexical overlap including minimum, maximum and mean across all clusters over time** are shown in Figure 7E and offer corpus-level insights. For example, the maximum and mean lexical overlap increases from the 1970s onwards, which may be a result of the improvement of OCR quality over time. On the basis of individual titles, it also shows that *Confédéré* defies this trend, with mean and maximum overlap constantly decreasing since the 1970s.

#### 4.1.4. Passages tab

The third tab titled *Passages* supports close reading of a given text reuse cluster (Task 2–Cluster overview). The list of passages can be sorted by date, lexical overlap, cluster size, time span and passage size. Figure 4D displays cluster c62714, which has a large time span of 19,188 days (ca. 52 years). A closer look reveals that it contains an article published in 1945 about the attack on Hiroshima, which was republished by multiple newspapers at the time and then rediscovered in 1998, when it was republished again, this time by *Gazette de Lausanne* and *Journal de Genève*.

Within the same tab, the *Compare* button below the snippet preview opens a comparison view (Task 3–Compare passages). Figure 4E highlights differences between two passages. Such differences can result from editorial interventions, including additions, and omissions, but also from OCR variation. Users select a “start passage” of interest, which appears on the left side, and can then cycle through all other passages in a cluster using arrow buttons on the right side. Characters that are only present in the start passage are highlighted in red, those that are only present in the compared passage are highlighted in green. Here we see a side-by-side view of two passages that cover protests by suffragette activists and an ensuing attack on Winston Churchill in 1910. On closer inspection, it also reveals interesting nuances in the coverage of the event: whereas the *Gazette de Lausanne* (left) does not make an explicit link between the assailant and the suffragettes, *L'independence luxembourgoise* (right) asserts that the attacker was believed to be part of the movement.

## 5. Evaluation

### 5.1. Evaluation setting

To evaluate the prototype, we invited various scholars to review the interface independently and remotely. We received 13 responses: 5 from (digital) historians with research experience in historical newspapers, 4 from computational linguists with experience in TRD, 3 from humanities scholars with experience in text reuse and virality, and 1 from a software developer with experience in text reuse visualization. Five of these evaluators had also participated in the workshop.

The evaluation was carried out by means of a form^14^ which contained five evaluation tasks that highlighted the different functionalities of the interface and were illustrated with instructive examples. They correspond to Task 1–Overview, Task 2–Cluster overview, Task 3–Compare passages, Task 5–Types, and Task 6–Research corpora.

Since the evaluation took place remotely, reviewers had to first familiarize themselves with the interface and the selected tasks. Therefore, for each evaluation task, the evaluators were first presented with the task definition (with an opportunity to comment) before being given instructions on specific operations to be tested in the interface, along with illustrative examples. For each evaluation task, the evaluators were asked to rate the difficulty of the task on a five-point scale. A concluding evaluation section allowed for an overall assessment of the interface, including its ability to effectively support the tasks presented, the quality of accompanying information, the ease of navigation, and the responsiveness of the system. Finally, evaluators were asked to indicate any irritations (“*Is there anything in the application that does not make sense? Does anything feel out of place?”*) and recommendations for its improvement (“*Future development of the application should focus on these tasks / features / overall improvements”*).

### 5.2. Discussion of evaluation results

**Evaluation tasks 1a-c: Obtain an overview of text reuse in a corpus, collection, or query**. This first segment familiarized evaluators with different aspects of the interface, notably search, filters, the tab views and the close-up view and was divided into three sub-tasks.^15^ Evaluators found the interface intuitive overall, but also confirmed that familiarity with text reuse concepts such as clusters or passages is an important prerequisite. Suggested improvements included the ability to compare the presence of text reuse in the entire corpus with the presence of text reuse discovered as a result of specific query in order to better contextualize the findings.

Especially for this first task, the feedback also reflected the learning experience of those evaluators who were using the *impresso* application for the first time. Critiques of individual interface components will be discussed below.

**Evaluation task 2: Obtain an overview of a single cluster**. The task was identified as part of an exploratory workflow: “*It seems a typical task again, like drilling down into a specific set of documents after first gathering a larger scale view in task 1”*. Regarding task implementation, evaluators found the interface to be “*convenient and intuitive”* and suggested high-level fingerprint views for (sets of) clusters to help with the assessment of cluster content.

**Evaluation task 3: Compare differences between passages within a cluster**. Evaluators pointed out that this task helps scholars to identify different ideological lines in newspapers, but also enables tool criticism. Overall, it complements distant reading operations: “*I feel this task foregrounds the complexities of text reuse that remain hidden to the viewer who only gazes at the high-level statistics.”* Another evaluator noted: “*Useful on how newspapers frame and present an event based on their ideological and political preference.”* In terms of implementation, evaluators appreciated the ease of use of the comparative view and suggested more abstract exploration of editorial practices and the ability to compare multiple passage simultaneously.

**Figure 9:**
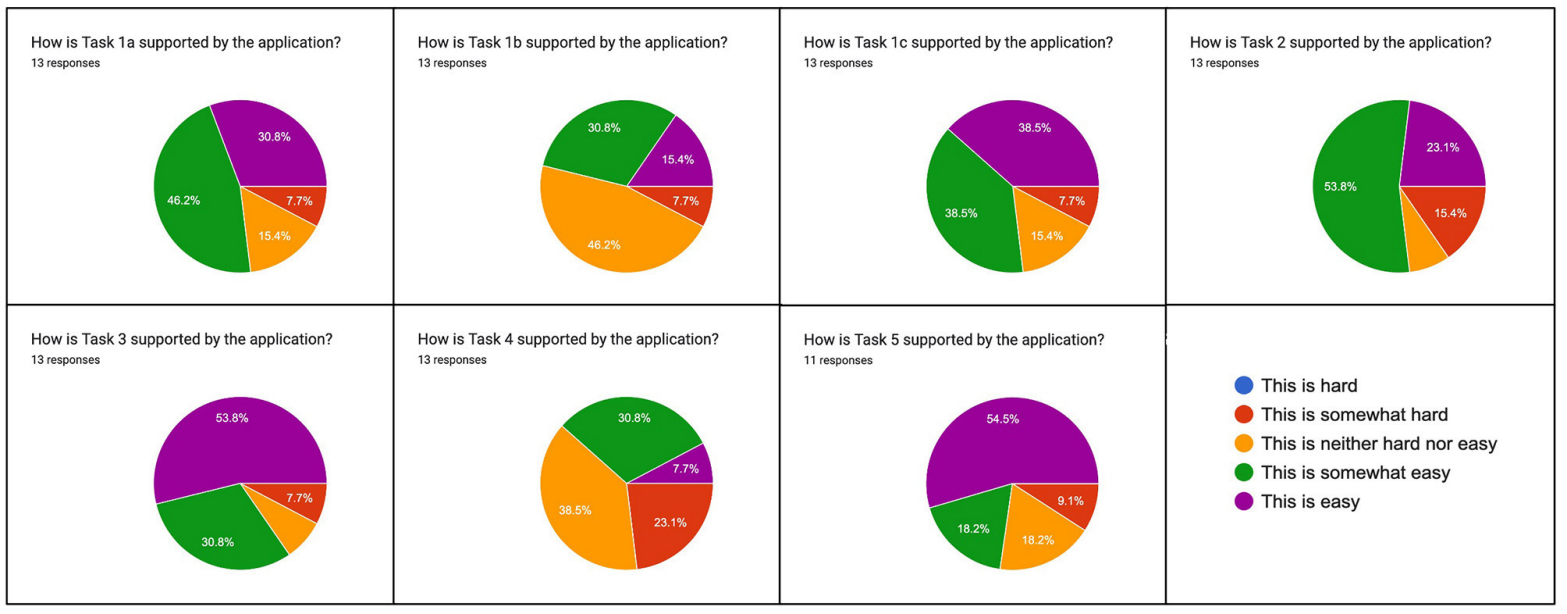
Rating of evaluation tasks regarding their perceived difficulty.

**Evaluation task 4: Identify different types of text reuse**.^16^ The addition of semantic levels to the exploration of text reuse data was overall welcomed. Task 4 was considered to be of particular interest to scholars. Evaluators noted that the gap between filtering operations and empirically observable types of text reuse had/has not yet been satisfactorily closed. One evaluator wrote: “*It seems be helpful to have some introduction to 1) a taxonomy of reuse types, and 2) the different kinds of phenomena and how each maps to various (meta)data variables.”* Feedback on task implementation was mixed, with a majority of evaluators finding the task as either “hard” or “somewhat hard” (Figure 9). Several evaluators suggested the creation of specific filters for empirically observed types of text reuse. This includes, for example, the reuse of older content by a newspaper title and explicit support to filter for cyclical reuse. Others, and this may echo the previous feedback, felt overwhelmed by the options to filter and visualize and did not know where to start. Still, others were content: “*Takes some getting used to the filters and functionalities, but nothing problematic.”*

**Evaluation task 5: Generate research corpora based on text reuse clusters**.^17^ This task received comparably little feedback since not all evaluators registered an *impresso* account in time to be able to test it. One evaluator called it useful for historians “*though there is a bit of a conceptual gap between reused passages and reused articles.”* Feedback on the implementation was generally positive, with some critiques of the slow speed of collection processing and of the difficulty of finding the data export function.

**Figure 10:**
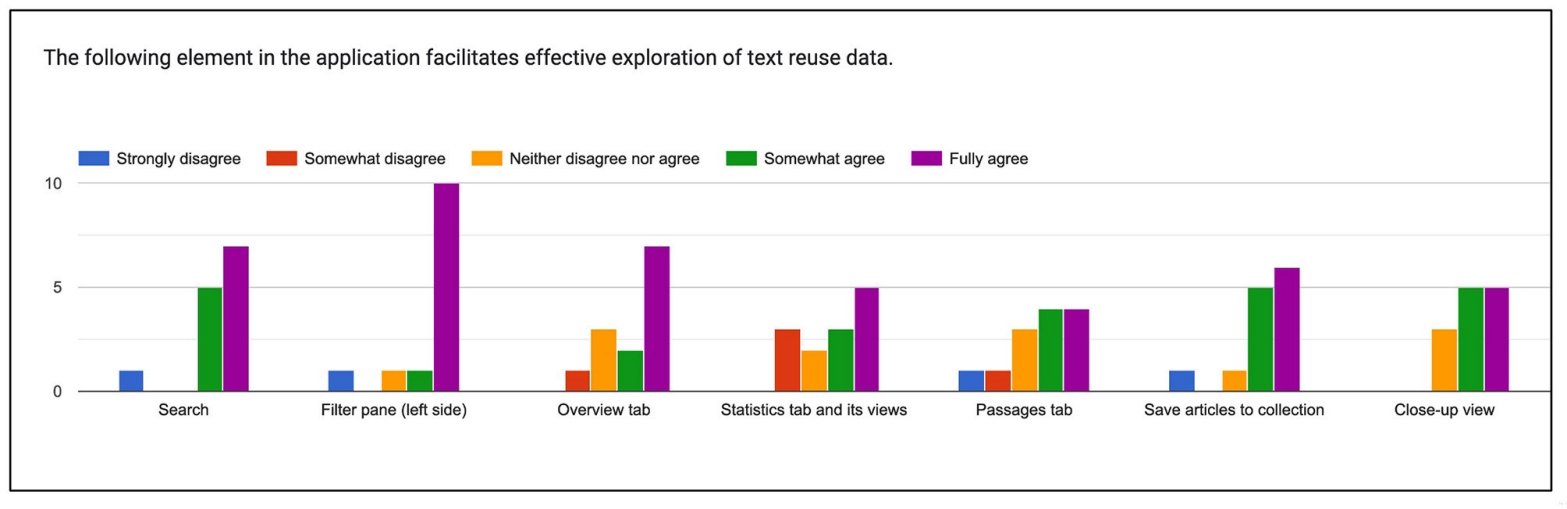
Rating of different components and views in the interface.

We move to the discussion of individual components within the interface:

**Search**. With one exception, all evaluators either “somewhat” or “fully agree” that the Search component facilitates effective exploration of text reuse data (Figure 10). We note, however, that previous experience with the *impresso* application was an advantage and that some evaluators who were new to it struggled at times, for example with searching for entities or the logic of removing filters.

**Filter pane**. Feedback was even more positive, but evaluators identified opportunities for improvement. These included the addition of units to histogram mouse-overs, a better indication that they are interactive, and pointers to a bug that prevented newspaper titles from being displayed as filter options.

**Overview tab**. Again, feedback was overwhelmingly positive (see Evaluation task 1, above). Critical remarks addressed its limited utility for exploring individual clusters, and the leap between text reuse passages and the display of article-level enrichments such as named entities or topics.

**Statistics tab**. Feedback revealed a need for more documentation and design improvements. Some evaluators struggled to read and interpret the charts, missed the option to zoom in on timelines, as well as more detailed information about their computation.

**Passages tab and passage comparison**. This segment divided evaluators. Some found it “*again easy and intuitive”* and “*Very user friendly, no remarks.”* Others missed a grouping of passages by cluster and struggled to find and operate the comparative view. Regarding the contrastive view, some found it difficult to cycle through different passages; they suggested changing the color scheme and eliminating some of the mismatches such as white space or OCR mistakes for easier viewing.

**Close-up view**. The close-up view was again rated positively, with the only criticism being the difficulty of finding it without direct instructions and a bug that prevented the display of passage previews.

**Figure 11:**
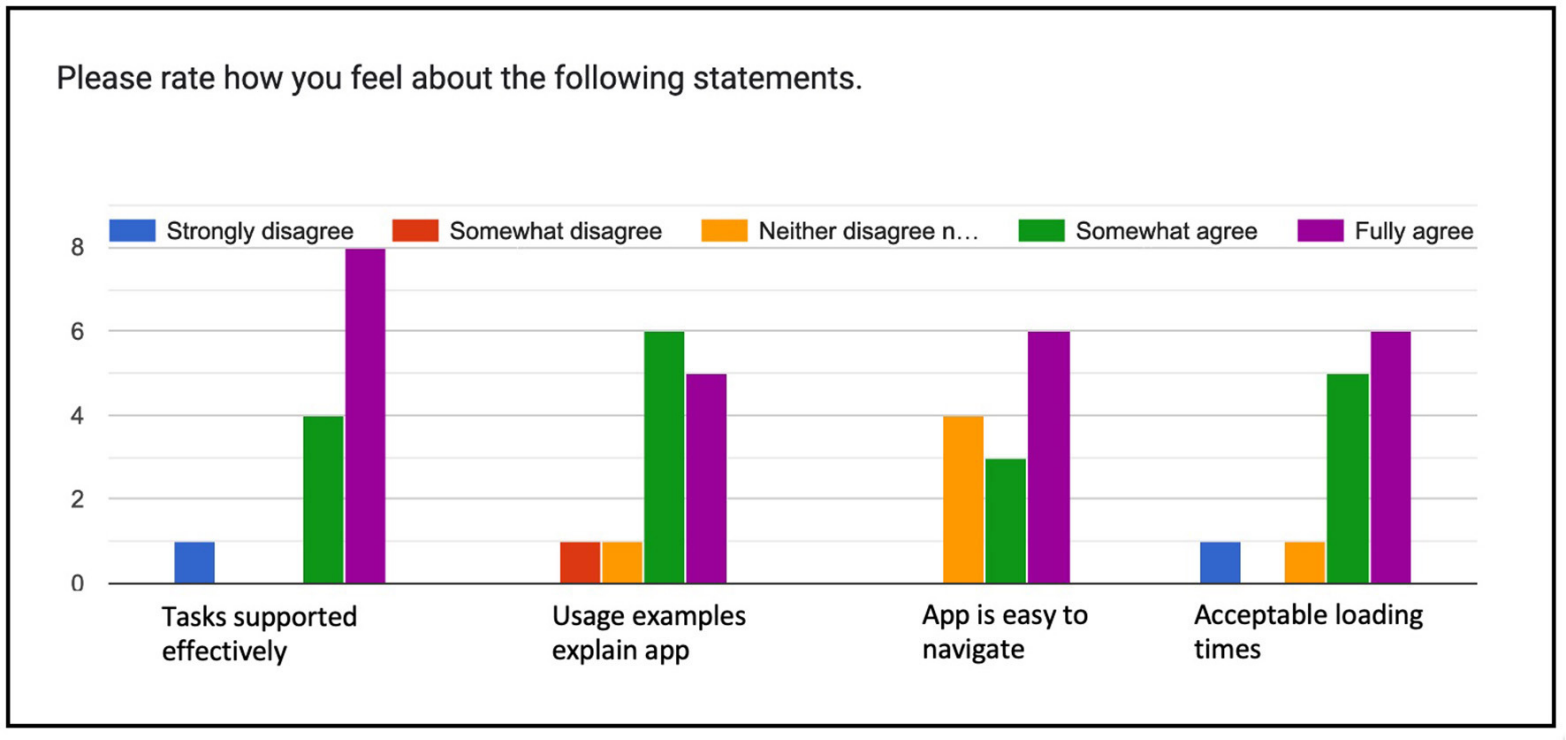
Overall rating of the interface.

In the overall rating of the interface (Figure 11), the vast majority of the 13 evaluators either “*somewhat”* or “*fully”* agreed that the interface supports the evaluation tasks (12), that the usage examples explained the interface (11), that it was easy to navigate (9), and that the loading times were acceptable (11). The interface clearly has a learning curve, as described by one evaluator: “*The functionality of the filters available here is impressive and of reasonable simplicity. I wouldn't describe it as ‘easy', mostly because there's a lot going on and a researcher not familiar with the dynamics of text reuse might be a bit lost, but I'm not sure I would trade the current depth of filtering for easier use.”*

The answers to our questions regarding irritations and future improvements confirm the critiques of the statistics tab and passages tab discussed above. At this stage of development, six evaluators found them “*either difficult to read or [they] did not provide useful insights.”* In addition, recommendations for future development addressed the already foreseen integration of *impresso*'s Inspect & Compare component (Düring et al., 2021) for side-by-side comparisons of article sets, higher speed for the creation of collections, API access to the data, and new filters based on a yet to be created taxonomy of text reuse types.

## 6. Conclusion and future work

In this paper, we have presented the prototype of the Text Reuse at Scale interface, to our knowledge the first interface to integrate text reuse data with other forms of semantic enrichment. We argue that it enables a versatile and scalable exploration of intertextual relations in historical newspapers. The interface was developed as part of the *impresso* project and combines powerful search and filter operations with close and distant reading perspectives. We reported on high-level research objectives and common user tasks for the analysis of historical text reuse data, and presented the interface together with the results of a user evaluation.

We have shown how the integration of text reuse data with semantic enrichment (content type, language, topics, named entities) has proved advantageous: firstly, as a means of effectively filtering for relevant sets of text reuse data; secondly, to help identify different types of text reuse; and thirdly, to provide overviews of the content of text reuse data. We have also demonstrated the interface's ability to retrieve text reuse based on temporal patterns, for example, distinguishing between content that spreads rapidly and news that is rediscovered after long time periods. Examples include the coverage of the bombing of Hiroshima, the reprinting of the same article on the event anniversary, and the reprinting of the 1945 article in 1998. Further, the interface reveals systematic co-publication patterns, independent of content, with the *Journal de Genève* and *Gazette de Lausanne* as the main examples. We have also shown its ability to give insights into text reuse cluster(s) based on topics, and to shift between distant and close reading operations. Finally, we have shown its usage for a critical assessment of corpora and variations in the performance of TRD based on the distributions of passages, cluster sizes, and lexical overlap over time.

At this stage of development, the Text Reuse at Scale interface supports many but not all of the previously discussed tasks for the exploration of historical text reuse data (see Table 2). Future development will address the integration with the *impresso* Inspect & Compare component to enable side-by-side comparisons of article sets which contain text reuse passages (in support of Task 4–Compare clusters and Task 7–Connections), and better support for temporal dimensions of text reuse data (Table 3). We will also take into account the need to improve the readability and documentation of the statistics tab and work to resolve the difficulties observed in the passages tab, together with minor bugs discovered during the evaluation.

The interface was developed to complement existing components in the *impresso* application for the exploration of semantically enriched historical newspapers. As such, it is tightly integrated with and dependent on the *impresso* infrastructure, its data and data models. Therefore, at this stage, the development of a standalone application suitable for text reuse data outside *impresso* is not foreseen.

Finally, text reuse detection tools to date still mostly operate at the “surface” level of language, i.e., they detect repeating patterns at the character and/or token level, but not at the semantic level. This means that they do not recognize translation as reuse. Recent advances in machine translation, as well as in multilingual language modeling and semantic indexing, may provide solutions in this direction and give an additional boost to research in transnational perspectives.

## Data availability statement

The raw data supporting the conclusions of this article will be made available by the authors upon request, without undue reservation.

## Author contributions

MD, MR, and DG contributed to the conception and design of the user workshop and developed the evaluation procedure. ME and MR revised the *impresso* technical infrastructure to suit the needs of the interface. DG was responsible for UI and UX development and integration into the *impresso* interface. MD and MR wrote the first draft of the manuscript. PA, KB, and BD wrote sections of the manuscript. All authors contributed to manuscript revision, read, and approved the submitted version.

## Footnotes

^1^ https://impresso-project.ch/

^2^Further information regarding the enrichment of the newspaper corpus can be found in the project blog (https://impresso-project.ch/blog/) and FAQ section (https://impresso-project.ch/app/faq).

^3^Content items refer to newspaper contents below the page level, such as articles, advertisements, images, tables, weather forecasts, obituaries, etc. For further details see: https://impresso-project.ch/news/2020/01/23/state-corpus-january2020.html.

^4^ https://impresso-project.ch/app

^5^ https://zenodo.org/communities/impresso/

^6^ https://www.etrap.eu/research/tracer/

^7^The Python package textreuse-blast provides an implementation of this method.

^8^ https://github.com/dasmiq/passim

^9^ https://graph-rare-books.ethz.ch/

^10^ http://networks.viraltexts.org/1836to1860/

^11^See Romanello and Hengchen (2020) for a list of available TRD software. Given the abundance of existing implementations, the lack of a systematic benchmark evaluation is clearly a major limitation in determining which tool is better suited for processing a specific type of corpus.

^12^Vesanto et al. (2017, p. 55) found that BLAST outperforms Passim in terms of recall when tested on a corpus characterized by extreme OCR noise.

^13^Discussions at the workshop also motivated work on a Master's thesis project which led to the successful detection of press agencies in the *impresso* corpus (Marxen, 2023) and the ability to filter for content from individual press agencies will be available in future versions of the interface.

^14^ https://zenodo.org/record/8009613/

^15^For the sake of simplicity, we combine feedback on task definitions and their implementation in the following paragraphs.

^16^Note that this evaluation task corresponds to Task 5–Types.

^17^Note that this evaluation task corresponds to Task 6 - Research corpora.
